# Prognostic significance of malnutrition risk in elderly patients with acute kidney injury in the intensive care unit

**DOI:** 10.1186/s12882-022-02949-7

**Published:** 2022-10-18

**Authors:** Na Wang, Ping Wang, Wen Li, Li Jiang, Meiping Wang, Bo Zhu, Xiuming Xi

**Affiliations:** 1 Emergency department of China rehabilitation research center, Capital Medical University, no.10 Jiaomen north Street, Fengtai District, Beijing, 100068 China; 2 Emergency department of Fu Xing Hospital, Capital Medical University, no. 20 Fuxingmenwai Street, Xicheng District, Beijing, 100038 China; 3 Department of Critical Care Medicine, Peking University Third Hospital, no. 49 Huayuan north Street, Haidian District, Beijing, 100191 China; 4 Department of Critical Care Medicine, Xuan Wu Hospital, Capital Medical University, no. 45 Changchun Street, Xicheng District, Beijing, 100053 China; 5 Department of Epidemiology and Health Statistics, School of Public Health, Capital Medical University, no.10 Xitoutiao, Youanmen, Fengtai District, Beijing, 100069 China; 6 Department of Critical Care Medicine, Fu Xing Hospital, Capital Medical University, no. 20 Fuxingmenwai Street, Xicheng District, Beijing, 100038 China

**Keywords:** Malnutrition risk, mNUTRIC, Elderly patient, AKI, Prognosis

## Abstract

**Background:**

Malnutrition is common in critically ill patients, but nutrition status in critically ill patients with acute kidney injury (AKI) has been poorly studied. Our study aimed to investigate the relationship between malnutrition risk and the occurrence and prognosis of AKI in elderly patients in the intensive care unit (ICU).

**Methods:**

Data were extracted from the Beijing Acute Kidney Injury Trial (BAKIT). A total of 1873 elderly patients were included and compared according to the clinical characteristics of AKI and non-AKI groups, and those of survivors and non-survivors of AKI in this study. Receiver operating characteristic (ROC) curves were used to analyse the predictive value of the modified Nutrition Risk in Critically Ill (mNUTRIC) score for the occurrence and 28-day prognosis of AKI. Multivariate Cox regression analysis was used to evaluate the effect of the mNUTRIC score on the 28-day mortality in AKI patients.

**Results:**

Compared with the non-AKI group, AKI patients had higher mNUTRIC scores, and non-survivors had higher mNUTRIC scores than survivors in AKI population. Moreover, multivariate Cox regression showed that 28-day mortality in AKI patients increased by 9.8% (95% CI, 1.018-1.184) for every point increase in the mNUTRIC score, and the mNUTRIC score had good predictive ability for the occurrence of AKI and 28-day mortality in AKI patients. The mortality of AKI patients with mNUTRIC > 4 was significantly increased.

**Conclusions:**

The elderly patients are at high risk of malnutrition, which affects the occurrence and prognosis of AKI. Adequate attention should be given to the nutritional status of elderly patients.

**Trial registration:**

This study was registered at www.chictr.org.cn (registration number Chi CTR-ONC-11001875) on 14 December 2011.

**Supplementary Information:**

The online version contains supplementary material available at 10.1186/s12882-022-02949-7.

## Background

Malnutrition is highly prevalent in the elderly population with acute kidney injury (AKI) [1, 2], which increases nosocomial mortality [3, 4]. Moreover, patients with malnutrition are proven to have an increased risk of AKI [5]. Nutritional status assessment is critical to identify elderly patients who may easily to suffer from AKI and are at risk of mortality [6]. Traditional nutritional screening tools, including weight loss, food intake reduction and laboratory values, are not reliable in AKI patients who cannot provide these details and may have water electrolyte disorders [7]. In addition, elderly patients with AKI in the intensive care unit (ICU) often suffer from volume resuscitation, resulting in rapid weight gain and even tissue edema. Body mass index, skin fold thickness and other data cannot accurately reflect the nutritional status of AKI patients.

The Nutrition Risk in Critically Ill (NUTRIC) score was proposed by Canadian scholar Heyland in 2011 [8], which is a nutritional assessment tool specifically designed for critically ill patients. The NUTRIC score includes 6 items: age, Acute Physiology and Chronic Health Evaluation II (APACHE II) score, Sequential Organ Failure Assessment (SOFA) score, number of comorbidities, length of hospital stay before admission to the ICU and interleukin-6 (IL-6) level. Each item is scored between minimum 0 and maximum 3 points according to its importance. When IL-6 cannot be obtained routinely, the modified NUTRIC (mNUTRIC) score is also acceptable [9]. Nutritional risk is low when the mNUTRIC score is 0 ~ 4, and high when the mNUTRIC score is 5 ~ 9 [8, 9]. The mNUTRIC score is related to adverse clinical outcomes (death and long duration of mechanical ventilation) [9–12].

Previous studies using albumin [13], prealbumin [14], body mass index (BMI) [15], Controlling Nutritional Status score (CONUT) [2], Nutritional Risk Screening 2002 (NRS-2002) [5, 6] and other indicators have found a higher mortality in AKI patients with malnutrition risk, but there is no report on the relationship between mNUTRIC score and AKI in the elderly population. The purpose of this study was to investigate the effect of the mNUTRIC score on the development and prognosis of AKI in the elderly ICU population.

## Materials and methods

### Study population

This study was a secondary analysis of the Beijing Acute Kidney Injury Trial (BAKIT) [16], which is a prospective, multicenter study that investigated the epidemiology of AKI in critically ill patients admitted to 30 ICUs at 28 tertiary hospitals in Beijing, China, from March 1 to August 31, 2012 (for a complete list of these hospitals and the personnel responsible for data collection, please refer to Additional file). Patients over 18 years old were enrolled consecutively, and only first-time ICU admissions were considered in this study. Patients with end-stage chronic kidney disease, renal replacement therapy (RRT) before admission to the ICU, renal transplantation in the previous 3 months, hospitalization less than 24 hours or incomplete clinical data were excluded. According to the World Health Organization standard, the elderly is defined as older than 60 years old [17].

### Data collection

Age, sex, BMI, admission date, admission diagnosis, comorbidities, organ failure, nephrotoxic drugs, baseline creatinine, APACHE II, SOFA, and the Simplified Acute Physiology Score II (SAPS II) score were recorded. Daily vital signs, laboratory data, urine output, use of vasoactive drugs, diuretics, and sepsis were continuously recorded for 10 days or until the patient was discharged from the ICU. The occurrence of AKI, length of mechanical ventilation (MV), RRT data and ICU length of stay (LOS) were also reported. The primary outcome was 28-day mortality.

We calculated the mNUTRIC score within the first day of ICU admission. Parameters for calculating the mNUTRIC score can be found in another article [8].

### Definition of AKI

AKI was defined and classified according to the Kidney Disease Improving Global Outcomes (KDIGO) guidelines [18]. The calculation of baseline creatinine can be found in our previous paper [16].

### Nutritional support

Nutritional support methods were based on the guidelines for enteral and parenteral nutrition issued by the Society of Critical Care Medicine (SCCM) and American Society for Parenteral and Enteral Nutrition (ASPEN) [19]. See our published article for details [12].

### Statistical analysis

SPSS software (IBM Corp., Statistics for Windows, version 22.0, Armonk, NY, USA) was used for data analysis, A two-sided *P* values < 0.05 was considered statistically significant. After normality testing, continuous variables were expressed as mean and standard deviation (SD) or medians (M) and quartiles (Q1, Q3), and compared using the Student’s t test or Mann-Whitney U test. Categorical variables were expressed as percentages, and the chi-squared test was used for comparison.

The hazards model (Cox) was used to analyse the risk factors for 28-day mortality in elderly patients with AKI. Since age, SOFA and APACHE II scores were included in the mNUTRIC score, collinearity analysis was required. Due to the collinearity of mNUTRIC with age or APACHE II, variables considered in the multivariate analysis included BMI, SAPS II, SOFA, mNUTRIC, sepsis, RRT, and AKI grades.

The discriminatory ability of the mNUTRIC score for AKI occurrence and prognosis was evaluated by receiver operating characteristic (ROC) curve analysis, and the areas under the curve (AUCs) were calculated. Youden index was used to establish the optimal cut-off value, and sensitivity, specificity, positive predictive value and negative predictive value were also reported. The Hosmer-Lemeshow goodness-of-fit test was used to test the calibration of the scoring system.

Kaplan-Meier survival curves were used to compare the cumulative survival rates among the four groups: low nutritional risk plus non-AKI vs. low nutritional risk plus AKI vs. high nutritional risk plus non-AKI vs. high nutritional risk plus AKI.

## Results

### Study population

**Fig. 1:**
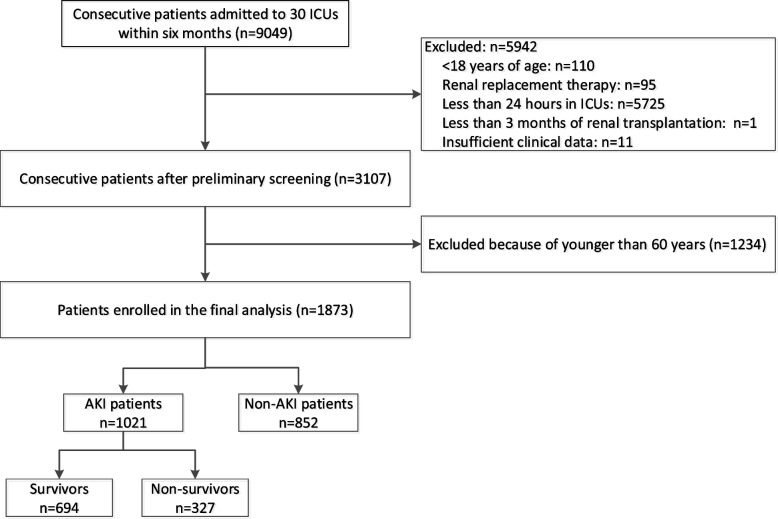
Flow chart of the study selection process

During the study period, 9049 patients were admitted consecutively, excluding unqualified cases, a total of 3107 patients were enrolled in the BAKIT study, and we previously published detail data on this population [16]. Of these patients, 1234 patients were excluded because they were younger than 60 years, leaving a final sample of 1873 patients for this study. A total of 1021 patients developed AKI, and 694 patients survived more than 28 days (Fig. 1). Among elderly patients with AKI, 333 were septic AKI, 331 were postoperative AKI, 85 were AKI due to hypovolemia, and 79 were AKI caused by drugs (including contrast agents). In the elderly population, 39.4% (738/1873) of patients had a higher nutritional risk according to the mNUTRIC score.

Baseline characteristics of the entire cohort and stratified data according to AKI occurrence are summarized in Table 1. The median age was 74 (Q1, Q3: 66, 81) years, and 60.4% were men. The all-cause 28-day mortality rate was 21.1% (396/1873), and the median ICU LOS was 5 (Q1, Q3: 2, 10) days. Among the included patients, the median BMI was 24 (Q1, Q3: 21, 26) Kg/m^2^, the median mNUTRIC score was 4 (Q1, Q3: 3, 5), the median APACHE II score was 16 (Q1, Q3: 12, 21), the median SAPS II was 38 (Q1, Q3: 30, 49) and the median SOFA score was 6 (Q1, Q3: 3, 9). MV was performed in 1215 (64.9%) patients, 802 patients (42.8%) were treated with vasopressors, and 180 patients (9.6%) underwent RRT.

**Table 1:** Patient characteristics by the occurrence of AKI

Characteristic	All patients (***n =*** 1873) M (Q1, Q3) Number (%)	AKI (***n =*** 1021) M (Q1, Q3) Number (%)	NonAKI (***n =*** 852) M (Q1, Q3) Number (%)	***P*** value
Age (years)	74 (66 -81)	75 (68 -82)	73 (65-79)	**< 0.001**
Male sex	1132 (60.4)	615 (60.2)	517 (60.7)	0.982
BMI	24 (21 -26)	24 (21 -26)	24 (21 -26)	0.981
**ICU course**				
Vasoactive therapy	802 (42.8)	439 (43.0)	363 (42.6)	0.985
MV	1215 (64.9)	687 (67.3)	528 (62.0)	0.056
Sepsis	628 (33.5)	472 (46.2)	156 (18.3)	**< 0.001**
**Severity of illness**				
mNUTRIC	4 (3-5)	5 (3-6)	3 (2-4)	**< 0.001**
APACHEII	16 (12-21)	18 (14-25)	13 (10-17)	**< 0.001**
SAPSII	38 (30-49)	43 (34-57)	33 (27-41)	**< 0.001**
SOFA	6 (3-9)	7 (4-10)	4 (2-7)	**< 0.001**
**Admission category**				
Medical	967 (51.6)	636 (62.3)	331 (38.8)	**< 0.001**
Surgical	906 (48.4)	385 (37.7)	521 (61.2)	
**Comorbidities**				
Hypertension	929 (49.6)	527 (51.6)	402 (47.2)	0.161
Coronary heart disease	550 (29.4)	335 (32.8)	215 (25.2)	**0.002**
Congestive heart failure	182 (9.7)	131 (12.8)	51 (6.0)	**< 0.001**
COPD	155 (8.3)	90 (8.8)	65 (7.6)	0.652
Diabetes	415 (22.2)	262 (25.7)	153 (18.0)	**< 0.001**
Chronic kidney disease	139 (7.4)	111 (10.9)	28 (3.3)	**< 0.001**
Liver disease	47 (2.5)	31 (3.0)	16 (1.9)	0.286
Cancer	372 (19.9)	200 (19.6)	172 (20.2)	0.951
**Category of ICU admission diagnosis**				
Cardiovascular	454 (24.2)	238 (23.3)	215 (25.2)	0.626
Respiratory	405 (21.6)	264 (25.9)	141 (16.5)	**< 0.001**
Neurologic	209 (11.2)	114 (11.2)	95 (11.2)	1.000
Trauma	113 (6.0)	51 (5.0)	62 (7.3)	0.121
Gastrointestinal	419 (22.4)	208 (20.4)	211 (24.8)	0.076
Kidney disease	91 (4.9)	62 (6.1)	29 (3.4)	**0.027**
Metabolic	28 (1.5)	17 (1.7)	11 (1.3)	0.810
**Outcomes**				
ICU LOS (days)	5 (2-10)	6 (3-12)	4 (2-7)	**< 0.001**
28-day mortality	396 (21.1)	327 (32.0)	69 (8.1)	**< 0.001**

Data are expressed as the median (interquartile range, IQR), and number (percentage). *BMI* Body mass index, *MV* Mechanical ventilation, *AKI* Acute kidney injury, *mNUTRIC* The modified Nutrition Risk in Critically Ill score, *APACHE II* Acute Physiology and Chronic Health Evaluation II, *SAPS II* Simplified Acute Physiology Score II, *SOFA* Sequential Organ Failure Assessment, *COPD* Chronic obstructive pulmonary disease, *LOS* Length of stay.

There were statistically significant differences in age, sepsis, mNUTRIC, APACHE II, SAPS II, SOFA, admission category, ICU LOS and 28-day mortality between AKI and non-AKI patients.

### Comparison of characteristics between survival and non-survival patients with AKI

Compared with the survivors, non-survivors were older, had lower BMI, higher nutritional risk, worse critical illness score, more use of mechanical ventilation and RRT, higher level of organ support, more prone to sepsis, and worse AKI grade. See Table 2. Among elderly patients with AKI, 54.0% (551/1021) were at higher nutritional risk according to the mNUTRIC score.

**Table 2:** AKI patients characteristics by 28-day mortality

Characteristic	AKI patients (***n =*** 1021) M (Q1, Q3) Number (%)	Survivors (***n =*** 694) M (Q1, Q3) Number (%)	Non-survivors (***n =*** 327) M (Q1, Q3) Number (%)	***P*** value
Age (years)	75 (68-82)	74 (67-81)	78 (70-83)	**< 0.001**
Male gender	615 (60.2)	420 (60.5)	195 (59.6)	0.967
Baseline creatinine (μmol/L)	82.0 (72.9-95.0)	81.0 (72.0-94.8)	84.0 (75.0-95.2)	0.774
BMI	24 (21-26)	24 (21-26)	23 (21- 25)	**0.012**
**Severity of illness**				
mNUTRIC	5 (3-6)	4 (3-6)	6 (5-7)	**< 0.001**
APACHEII	18 (14-25)	16 (12-21)	24 (18- 30)	**< 0.001**
SAPSII	43 (34-57)	39 (31-49)	55 (43-69)	**< 0.001**
SOFA	7 (4-10)	6 (4-9)	9 (6-12)	**< 0.001**
**ICU course**				
Vasoactive therapy	439 (43.0)	301 (43.4)	138 (42.2)	0.940
MV	687 (67.3)	452 (65.1)	235 (71.9)	0.101
sepsis	472 (46.2)	240 (34.6)	232 (70.9)	**< 0.001**
Staging of AKI 1	450 (44.1)	373 (53.7)	77 (23.5)	
2	246 (24.1)	168 (24.2)	78 (23.9)	**< 0.001**
3	325 (31.8)	153 (22.1)	172 (52.6)	
RRT	174 (17.0)	77 (11.1)	97 (29.7)	**< 0.001**
Organ support				
single organ	477 (46.7)	340 (49.0)	137 (41.9)	
dual organ	332 (32.5)	212 (30.5)	120 (36.7)	**< 0.001**
multiorgan	53 (5.2)	22 (3.2)	31 (9.5)	
**Outcomes**				
ICU LOS (days)	6 (3-12)	5 (3-12)	6 (4-11)	0.722

Data are expressed as the median (interquartile range, IQR), and number (percentage). *AKI* Acute kidney injury, *BMI* Body mass index, *mNUTRIC* The modified Nutrition Risk in Critically Ill score, *APACHE II* Acute Physiology and Chronic Health Evaluation II, *SAPS II* Simplified Acute Physiology Score II, *SOFA* Sequential Organ Failure Assessment, *MV* Mechanical ventilation, *RRT* Renal replacement therapy, *LOS* Length of stay.

### The predictive ability of mNUTRIC for the occurrence of AKI and poor outcomes

**Fig. 2:**
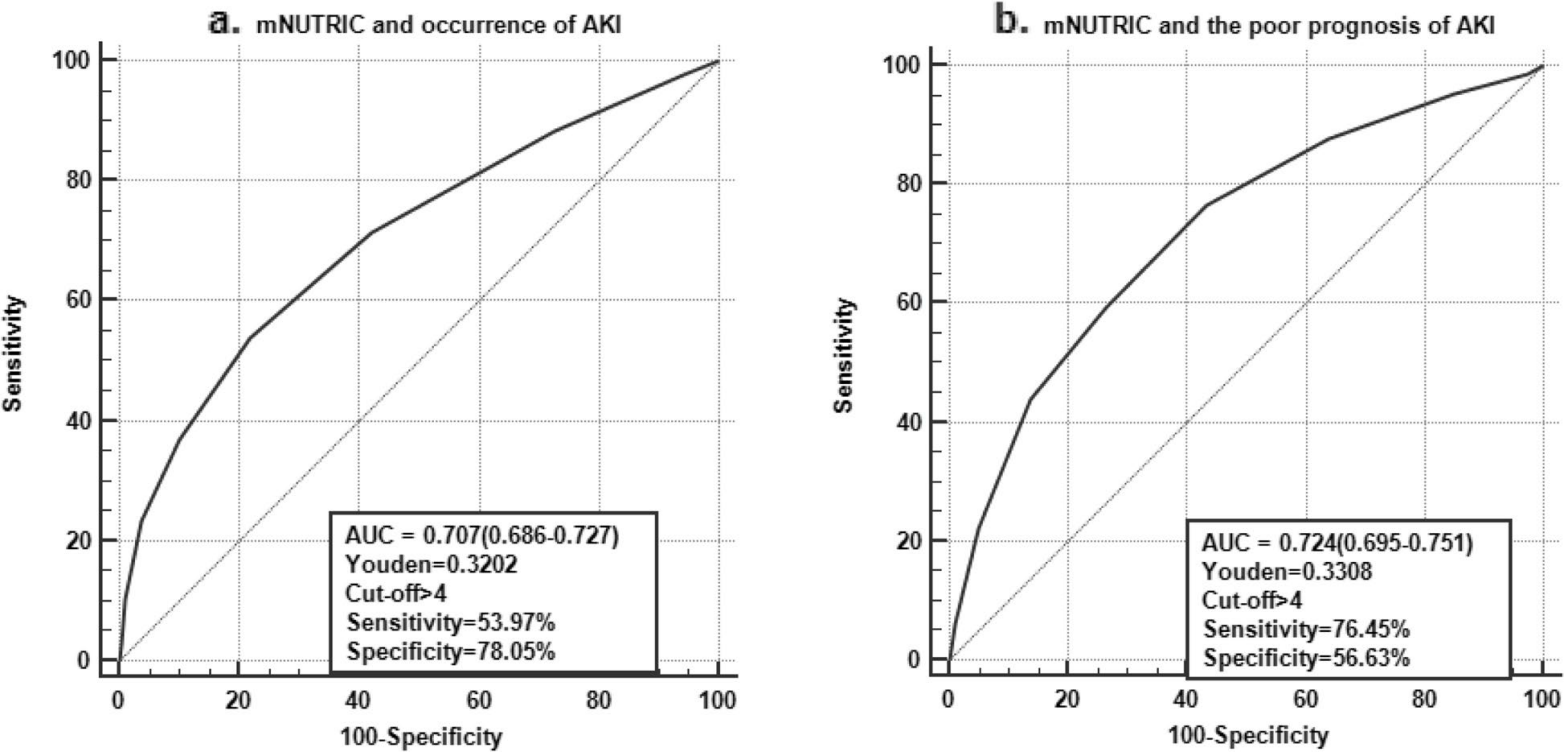
**a** ROC curves of mNUTRIC score for the occurrence of AKI in old patients. **2b** ROC curves of mNUTRIC score for 28-day mortality in old patients with AKI

The ROC curve of the mNUTRIC score predicting AKI occurrence is shown in Fig. 2a. The cut-off value was > 4 with a sensitivity of 53.97% and specificity of 78.05%. The ROC curve of the mNUTRIC score for 28-day mortality in AKI patients is shown in Fig. 2b. The cut-off value was > 4 with a sensitivity of 76.45% and specificity of 56.63%. The Hosmer-Lemeshow test showed that the goodness of fit of the score was good, and the chi-square (*P* value) values were 7.753 (0.170) and 3.260 (0.660), respectively. Patients with high nutritional risk had a higher incidence of AKI and a poorer prognosis than those with low nutritional risk (Table 3).

**Table 3:** Clinical events in patients with different nutritional risks

Events	All patients	Low nutrition risk	High nutrition risk	***P*** value
	(***n =*** 1873)	(mNUTRIC ≤ 4, ***n =*** 1135)	(mNUTRIC > 4, ***n =*** 738)	
AKI	1021 (54.5%)	470 (41.4%)	551 (74.7%)	**< 0.001**
28-day death	396 (21.1%)	113 (10.0%)	283 (38.3%)	**< 0.001**
In-hospital death	412 (22.0%)	105 (9.3%)	307 (41.6%)	**< 0.001**
RRT	180 (9.6%)	40 (3.5%)	140 (19.0%)	**< 0.001**

*mNUTRIC* The modified Nutrition Risk in Critically Ill score, *AKI* Acute kidney injury, *RRT* Renal replacement therapy.

### Cox regression analyses of 28-day mortality in AKI patients

A Cox regression model was used to test the effect of the mNUTRIC score in predicting 28-day mortality in patients with AKI (Table 4). Because the mNUTRIC score is collinear with APACHE II or age, variables considered for Cox regression analysis included BMI, mNUTRIC score, SAPS II, SOFA, sepsis, RRT and AKI grade. Multivariable analysis showed that 28-day mortality increased by 9.8% (95% CI, 1.018-1.184) for every point increase in the mNUTRIC score. In addition to mNUTRIC, SAPS II, sepsis, and worse AKI grade were significantly associated with a higher risk of death in multivariable analysis.

**Table 4:** Multivariate Cox regression analysis of 28-day mortality in AKI patients

Characteristic	Hazard ratio	95%CI	P
SAPSII	1.026	1.018-1.034	**0.000**
Sepsis	1.789	1.380-2.319	**0.000**
mNUTRIC	1.098	1.018-1.184	**0.015**
AKI grade	1.515	1.320-1.739	**0.000**

*mNUTRIC* The modified Nutrition Risk in Critically Ill score, *SAPS II* Simplified Acute Physiology Score II, *AKI* Acute kidney injury, *CI* Confidence interval.

### Survival curve of 28-day mortality by the low or high of mNUTRIC scores in the AKI/non-AKI patients

**Fig. 3:**
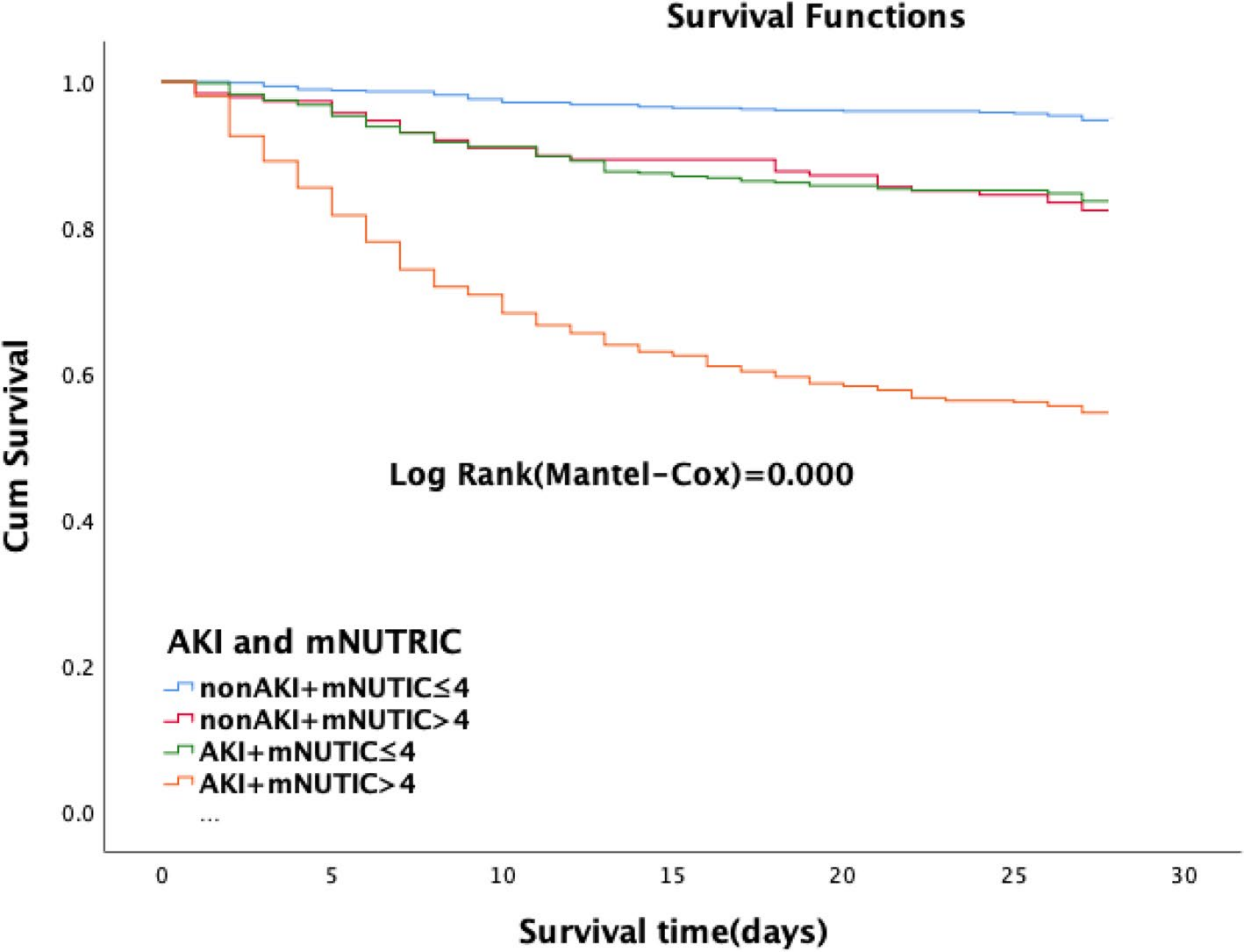
Survival curve of 28-day mortality by the low or high mNUTRIC score in the AKI/non-AKI patients

**Fig. 4:**
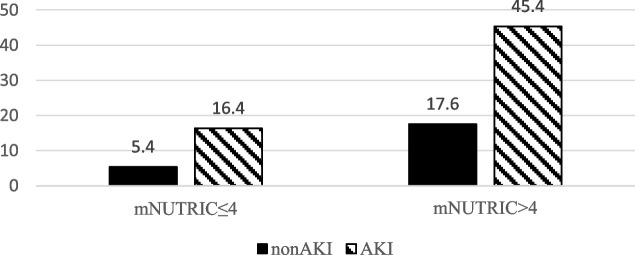
28-day mortality of AKI/non-AKI patients by the mNUTRIC score

The patients were divided into four groups according to the cut-off value of the mNUTRIC score and the presence or absence of AKI: low nutritional risk plus non-AKI vs. low nutritional risk plus AKI vs. high nutritional risk plus non-AKI vs. high nutritional risk plus AKI. Kaplan-Meier survival curves were used to compare the cumulative survival rates among the four groups (Fig. 3). The 28-day survival rate was significantly increased in patients with high nutritional risk and AKI. When patients had AKI and high nutritional risk, the 28-day mortality increased to 45.2% (Fig. 4).

## Discussion

Due to the inflammatory response, surgery, trauma and other reasons, catabolism is significantly enhanced and anabolism is weakened in critically ill patients, resulting in increased nutritional risk. Critically ill patients experience the blow of the disease, and their immune function is suppressed. When combined with malnutrition, immune suppression is further aggravated, resulting in aggravated infection, delayed wound healing, acquired muscle weakness and difficulty in weaning, resulting in increased complications, including AKI and increased mortality. Malnutrition is common in the elderly population [20, 21], energy intake decreases as the body weakens with age, and age is an important factor for malnutrition. Some nutrition screening tools, such as NRS-2002 [22], Patient-Generated Subjective Global Assessment (PG-SGA) [23] and mNUTRIC score, all include age. Malnutrition is often found in patients with acute kidney injury (AKI) [3, 5, 24], and it is an independent risk factor for poor prognosis in critically ill patients [25, 26]. It affects the occurrence and development of AKI independently of non-nutritional factors, increases in-hospital mortality, prolongs hospitalization time and increases hospitalization expenses [5].

The European Society for Clinical Nutrition and Metabolism (ESPEN) guidelines recommended that all hospitalized patients with AKI should be screened for malnutrition [27]. However, due to the complex and multifactorial nature of malnutrition in patients with kidney diseases, the best tool to identify patients at high risk of malnutrition is still in dispute [1, 28]. The NUTRIC score was designed for the ICU population, and its performance for critically ill patients may be better than NRS-2002 [28, 29]. Our study used the mNUTRIC score as an assessment tool to evaluate the nutritional risk of elderly AKI patients and found that 551 (54.0%) of these patients had a higher nutritional risk in elderly AKI population. Our findings support the need to enhance the identification of malnutrition risk among elderly patients in the ICU. This may improve the risk stratification of patients and guide the prevention of AKI.

Our study found that older patients with higher nutritional risk were more likely to develop AKI than those with lower nutritional risk (74.7% vs. 41.4%) (Table 3). The predictive ability of the mNUTRIC score for the occurrence of AKI was good, but its sensitivity was low (Fig. 2). Similarly, another study showed that increased nutritional risk was independently associated with the presence of contrast-induced AKI (CI-AKI), and malnutrition assessment of elderly patients before diagnosis or coronary intervention may help clinicians identify patients with elevated risk for CI-AKI [1]. Wei et al. also found that moderate-severe malnutrition evaluated by the CONUT score is associated with a higher risk of contrast-associated AKI (CA-AKI) in elderly patients undergoing percutaneous coronary intervention (PCI) [2]. Recently, a retrospective propensity score matching study enrolled 46,549 inpatients and found that patients with NRS-2002 scores ≥3 had a higher incidence of AKI than normal nutritional patients, and the undernourished patients who developed AKI had a far worse prognosis than normal nutritional patients [5]. Early identification of patients with high nutritional risk and adequate nutritional support treatment to reduce the occurrence of AKI is very important to improve the prognosis of patients.

Malnutrition is common in critically ill patients and is closely related to the prognosis of AKI patients [13, 14]. However, the nutritional status of AKI patients is often ignored [21]. Accurately assessing the nutritional status of patients and providing nutritional support is still a challenging task in AKI treatment. Fiaccadori et al. conducted a study of 309 patients with AKI and found that 58% of patients had malnutrition, and severe malnutrition was associated with poor prognosis [3]. Another study also found that low calorie intake, high C-reactive protein level, edema and low nitrogen balance were significantly associated with the risk of death in AKI patients [4]. The risk of malnutrition assessed by the NRS-2002 helps to identify high-risk patients with AKI and mortality, and patients with acute coronary syndrome can benefit from further nutritional intervention and prevention of AKI [6]. A meta-analysis showed that protein-energy wasting (PEW) assessed using subjective global assessment (SGA) was associated with greater mortality risk (RR: 1.99, 95% CI: 1.36–2.91). Individual nutrition parameters, such as serum chemistry, body mass, muscle mass, and dietary intake, were not consistently associated with mortality in patients with AKI [30]. Our study showed that the mNUTRIC score was an effective tool to evaluate the prognosis of AKI patients. After adjusting for multiple risk factors, 28-day mortality in AKI patients increased by 9.8% (95% CI, 1.018-1.184) for every point increase in the mNUTRIC score.

Our study found that high nutritional risk patients assessed using the mNUTRIC score had a worse prognosis than low nutritional risk patients. When AKI was present, the mortality increased significantly (Fig. 3 and 4), which is consistent with other studies [5, 6, 31]. Under pathological conditions, the interaction between malnutrition and AKI is close and complex. For example, malnutrition may lead to AKI, which in turn is a harmful factor of malnutrition. Our research showed that high nutritional risk is closely related to AKI, and both contribute to the poor prognosis of patients. Li et al. [5] also found that there was a strong association between the NRS-2002 and AKI and that the risk of AKI changed in parallel with the NRS-2002 score. Both AKI and NRS-2002 scores ≥3 can worsen the prognosis.

Our study had several limitations: First, our investigation was limited to the risk factors available in the original database and did not record albumin, prealbumin, or total cholesterol, etc., although serum markers may not have good predictive ability [32]. Second, we did not differentiate the onset and duration of AKI, which may affect patient outcomes. Third, there was no dynamic nutritional assessment, which might have been more meaningful. Further studies are needed to determine the value of high nutritional risk in elderly patients with AKI.

## Conclusions

High nutritional risk based on the mNUTRIC score is common in elderly patients with AKI and is a risk factor in poor prognoses. Early nutritional risk assessment and targeted treatment are recommended for critically ill patients, which may be of great significance in preventing the occurrence of AKI and improving the prognosis of patients.

## Supplementary Information

**Additional file 1.**

## Data Availability

The datasets generated and analysed during the current study are not publicly available due to there was a confidentiality agreement signed with the partner hospitals, but available from the corresponding author on reasonable request.

## Abbreviations

AKI: Acute kidney injury
ICU: Intensive care unit
BAKIT: The Beijing Acute Kidney Injury Trial
ROC: Receiver operating characteristic curve
mNUTRIC: The modified Nutrition Risk in Critically Ill score
APACHE II: Acute Physiology and Chronic Health Evaluation II
SAPS II: Simplified Acute Physiology Score II
SOFA: Sequential Organ Failure Assessment
BMI: Body mass index
CONUT: Controlling Nutritional Status score
RRT: Renal replacement therapy
MV: Mechanical ventilation
LOS: Length of stay
KDIGO: Kidney Disease Improving Global Outcomes guidelines
COPD: Chronic obstructive pulmonary disease
CI: Confidence interval
AUC: The areas under the curve
PG-SGA: Patient-Generated Subjective Global Assessment
ESPEN: The European Society for Parenteral and Enteral Nutrition
PEA: Protein-energy wasting
